# Enhancer of Acetyltransferase Chameau (EAChm) Is a Novel Transcriptional Co-Activator

**DOI:** 10.1371/journal.pone.0142305

**Published:** 2015-11-10

**Authors:** Takeya Nakagawa, Tsuyoshi Ikehara, Masamichi Doiguchi, Yuko Imamura, Miki Higashi, Mitsuhiro Yoneda, Takashi Ito

**Affiliations:** Department of Biochemistry, Nagasaki University School of Medicine, Nagasaki, 852–8523, Japan; St. Georges University of London, UNITED KINGDOM

## Abstract

Acetylation of nucleosomal histones by diverse histone acetyltransferases (HAT) plays pivotal roles in many cellular events. Discoveries of novel HATs and HAT related factors have provided new insights to understand the roles and mechanisms of histone acetylation. In this study, we identified prominent Histone H3 acetylation activity *in vitro* and purified its activity, showing that it is composed of the MYST acetyltransferase Chameau and Enhancer of the Acetyltransferase Chameau (EAChm) family. EAChm is a negatively charged acidic protein retaining aspartate and glutamate. Furthermore, we identified that Chameau and EAChm stimulate transcription *in vitro* together with purified general transcription factors. In addition, RNA-seq analysis of Chameu KD and EAChm KD S2 cells suggest that Chameau and EAChm regulate transcription of common genes *in vivo*. Our results suggest that EAChm regulates gene transcription in *Drosophila* embryos by enhancing Acetyltransferase Chameau activity.

## Introduction

The eukaryotic genome is packaged into a higher-order nucleoprotein structure called chromatin that can be replicated and segregated appropriately during the cell cycle. Transcriptional regulation from chromatin is a dynamic and precise process through its condensation and de-condensation accompanied by histone modification [1]. The basic unit of chromatin is called the nucleosome, composed of an octamer of core histones (two each of H2A, H2B, H3 and H4), around which 146 base pairs of DNA are wrapped [2]. Nucleosomes act as general repressors of basal transcription, inhibiting transcriptional initiation and elongation by RNA pol II [3]. Transcription from the repressed templates is activated by the actions of several nuclear factors, such as sequence-specific DNA-binding transcriptional activators, chromatin remodeling complexes, and histone acetyltransferases.

Recently, diverse posttranslational modifications of nucleosomal histones have been identified, including methylation, phosphorylation, ubiquitylation, acetylation and sumoylation [4–6]. Among these posttranslational modifications, histone acetylation is the best characterized histone modification, because of the importance of regulation of chromatin structure and gene transcription. Histone acetyltransferase (HAT) is defined as an enzyme that acetylates core histones. The study of HATs has advanced rapidly, because a number of HATs have been isolated from various organisms and these studies demonstrated that HATs are evolutionarily conserved from yeast to humans, and HAT complexes often have multiple subunits [7–9]. Historically, HATs are grouped into two general classes based on their intracellular location and substrate specificity as either nuclear A-type (HAT A) or cytoplasmic B-type (HAT B)[10]. However, some HAT proteins may function in multiple complexes or locations and thus not precisely fit these two classifications [11].

Among A-type HATs, the largest histone acetyltransferase family is the MYST family comprised of three subgroups; the Moz /Qkf, Tip60/Mof, and Hbo1/ Chameau subfamilies [12, 13]. MYST histone acetyltransferases are defined by their MYST domain containing an acetyl-CoA binding site and a C2HC-type zinc finger. Despite their structural similarities, the function and biological properties of MYST proteins are diverse including gene transcription, DNA damage repair and replication. Importantly, regulation of MYST histone acetytransferase needs to be understood [12].

In this study, we found prominent nucleosomal histone H3 acetylation activity in *Drosophila* embryo extracts. We purified its activity and showed that prominent histone H3 acetylation activity composed of MYST histone acetytransferase Chameau and Enhancer of Acetyltransferase Chameau (EAChm), stimulated transcription *in vitro*.

## Results

### Prominent Histone H3 acetyltransferase activity is composed of Chameau and Enhancer of Acetyltransferase Chameau (EAChm)

**Fig 1:**
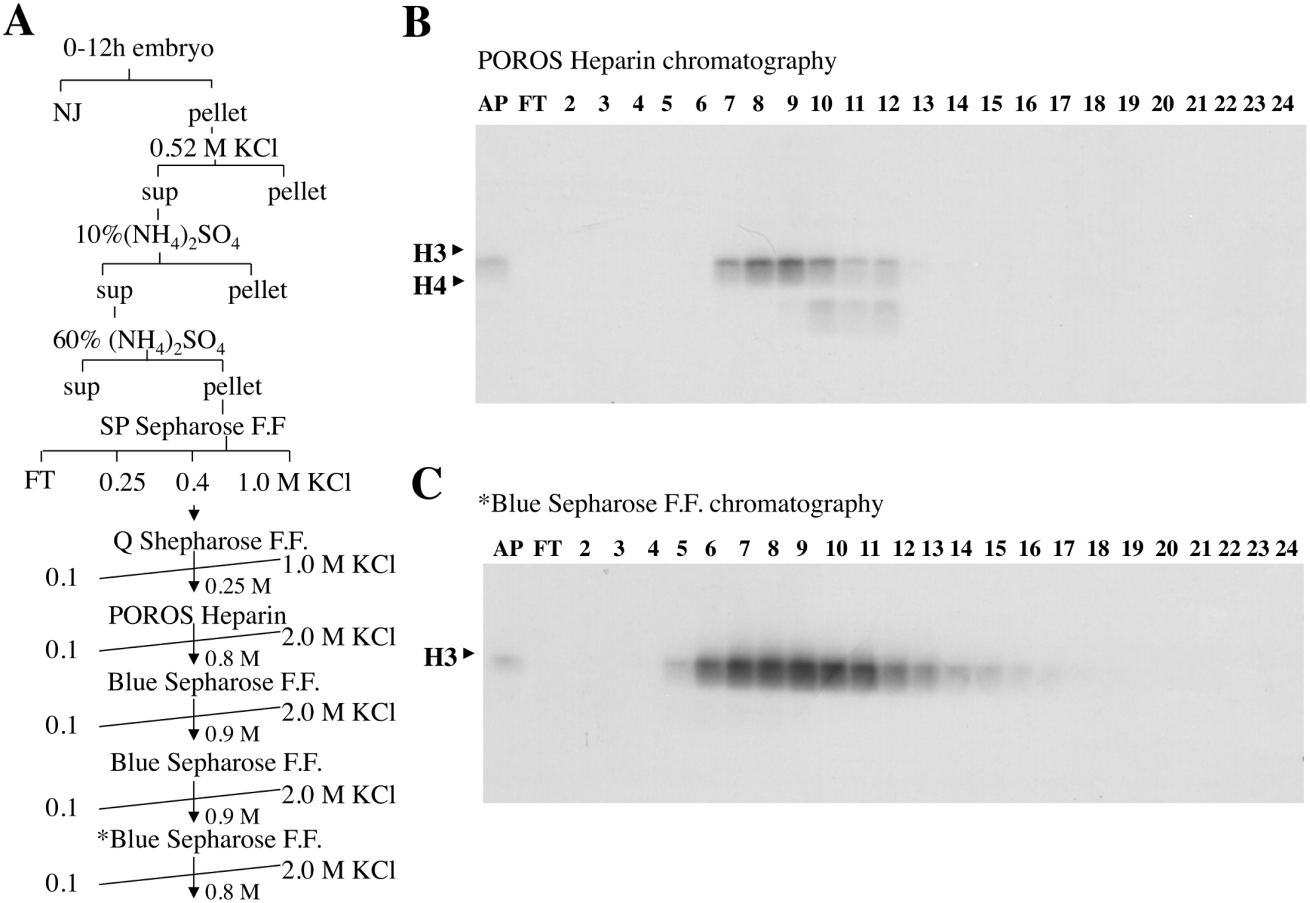
Purification of Histone H3 acetyltransferase. A) Scheme for purification of histone H3 acetyltransferase. Histone H3 acetyltransferase was identified in 0–12 hour Drosophila embryos using column chromatography. Elution conditions for each column chromatography step are indicated. B), C) Histone acetylation activity was monitored by HAT assay. Fractions were incubated with salt dialyzed chromatin as templates in the presence of [3H] acetyl-CoA. After incubation, the reaction mixtures were run on 13.5% SDS-PAGE and acetylated histones incorporating ^3^H were detected by autoradiography. B) HAT assay from POROS Heparin chromatography in Fig 1A and 1C) HAT assay from *Blue Sepharose F.F. chromatography in Fig 1A. “NJ”: Nucleic Juice is a nuclear extract. “AP”: applied sample that is loaded for column chromatography. “FT”: Flow through is the unbound fraction during column chromatography.

Histone acetylation in the nucleus plays pivotal roles in chromatin assembly and gene transcription, yet the mechanisms that regulate the acetyltransferase activity and HAT complex assembly have not been fully determined. Since we identified prominent histone H3 acetylation activity in nuclear extracts made from 0–12 hour Drosophila embryos, we started to purify the activity to uncover its regulation mechanisms. To monitor histone acetylation activity, we added [3H] acetyl-CoA to the reaction as well as salt dialyzed chromatin and extracts or fractions. As depicted in Fig 1A, we purified the activity using column chromatography. We detected histone H3 acetyl-transferase activity in fractions from POROS Heparin chromatography together with weak H4 acetyl-transferase activity (Fig 1B). The fractions that contained the histone H3 acetyltransferase activity were further purified to exclude contaminating H4 acetyltransferase activity. H3 acetyltransferase activity was successfully enriched in the fractions from subsequent Blue Sepharose chromatography (Fig 1C).

**Fig 2:**
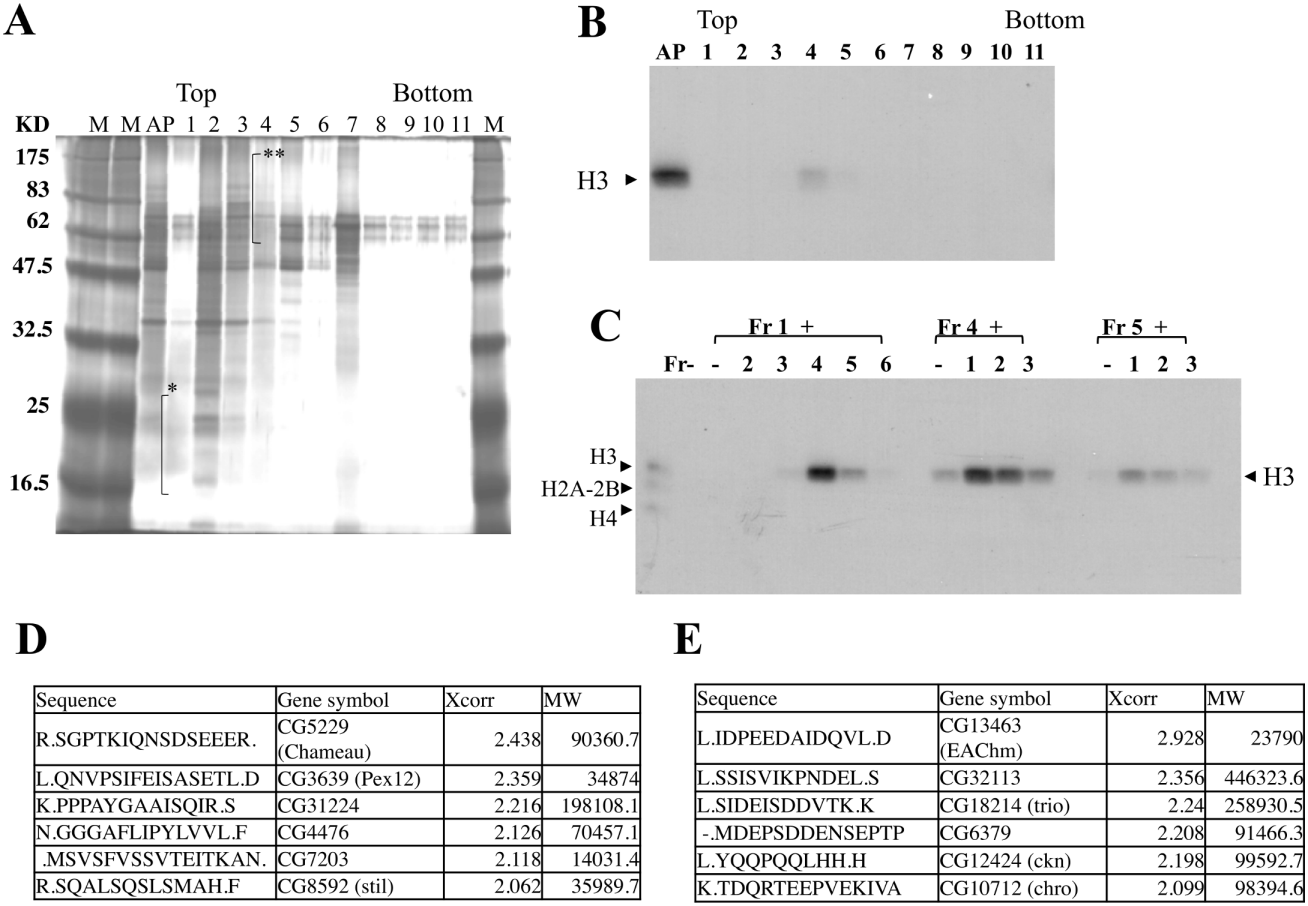
Histone H3 acetyl transferase activity is composed of Chameau and Enhancer of Acetyltransferase Chameau (EAChm). A) Silver staining of fractions from glycerol gradient sedimentation. The Blue sepharose fraction was further purified by glycerol gradient sedimentation (BECKMAN SW60 rotor, 60,000 rpm for 20 hours). All fractions were analyzed by silver staining. Chameau and Enhancer of Acetyltransferase Chameau (EAChm) were identified by LC-MS/MS analysis of gel fragments indicated with (**) and (*), respectively. B), C) HAT assay using single fraction or combination of the fractions from glycerol gradient sedimentation as indicated. After incubation in the presence of [3H] acetyl-CoA, the reaction mixtures were run on 13.5% SDS-PAGE and analyzed by autoradiography. D, E) Table of identified peptides from the gel fragments (**: D, *: E) by LC-MS/MS analysis. These tables show the identified peptide sequence, reference gene ID (gene symbol), Xcorr score and molecular weight.

To further purify the HAT activity, the fractions from the Blue Sepharose chromatography were applied to glycerol gradient sedimentation and subsequently analyzed by SDS-polyacrylamide gel electrophoresis (SDS-PAGE) with silver staining (Fig 2A). Fig 2B shows histone acetylation activity of glycerol gradient sedimentation fractions. First, we focused on fraction 4 since it had weak activity. A glycerol gradient usually dilute samples 1:2~1:3. However, activity is lost about 1:7 according to measurements made using a densitometer. Thus, we speculate that H3 acetyltransferase activity is composed of multiple factors. We performed assay by comparing the activity in fractions and found some activity in fraction 4 in the preliminary evaluation. In addition we done assay by combination of the fraction in addition to fraction 4. Finally we identified that activity is composed of fraction 1 and 4. These results suggested that the enhancing factor of the H3 acetyl-transferase activity is present in this fraction. To identify H3 acetyltransferase and its enhancing factor, fractions from the glycerol gradient sedimentation step were subjected to LC-MS/MS analysis. Since obvious bands were not observed, we performed mass analysis using broad SDS gel fragments. To identify H3 acetyltransferase, the peptide sequence (R.SGPTKIQNSDSEEER.) was obtained from the SDS gel fragments indicated by (**) in Fig 2A and found to be identical to segments of *Drosophila* CG5229 gene product that is known as the histone acetyltransferase, Chameau (Fig 2D). There was no other peptide predicted to be histone acetyltransferase other than Chameau. For the enhancing factor of H3 acetyltransferase activity, the peptide sequence (L.IDPEEDAIDQVL.D) was obtained from the SDS gel fragments indicated by (*) in Fig 2A and found to be identical to segments of *Drosophila* CG13463 gene product (Fig 2E). CG13463 is a protein coding gene but its molecular function was unknown (see FlyBase report; http://flybase.org). Since CG13463 gene product was the only peptide which shows a high Xcorr score and proper molecular weight in the top fraction of glycerol gradient sedimentation, we tentatively named this gene Enhancer of Acetyltransferase Chameau (EAChm).

### Purified recombinant EAChm stimulates acetyltransferase activity of Chameau

**Fig 3:**
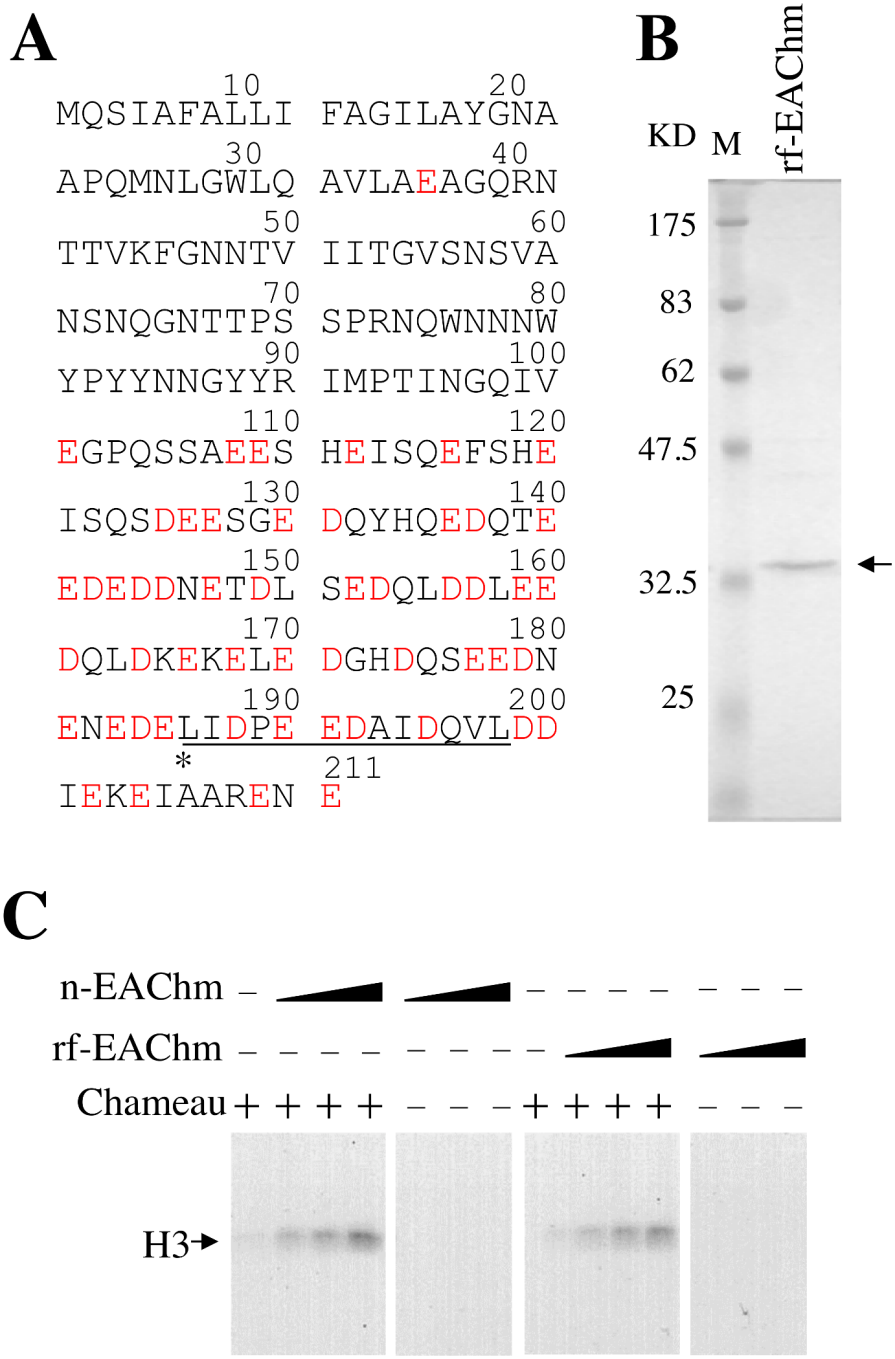
Purified recombinant EAChm stimulates acetyltransferase activity of Chameau. A) Amino acid sequence of EAChm. Acidic residues D and E are indicated with a red letter. The peptide sequence identified by mass spectrometry is underlined. B) Expression of flag-tagged EAChm in *E*.*coli*. After purification, protein was subjected to 10% SDS—PAGE and then visualized by Coomassie brilliant blue R staining. C) Recombinant flag-tagged EAChm (rf-EAChm) enhances H3 acetyl transferase activity. HAT assay was performed in the presence of either native EAChm (n-EAChm) or rf-EAChm, Chameau (fraction 4 from glycerol gradient sedimentation) and [3H] acetyl-CoA using salt dialyzed chromatin.

*Drosophila* CG13463 gene product is an acidic protein consisting of 211 amino acids containing glutamate and aspartate in its c-terminus as shown in Fig 3A. To examine if the gene product of EAChm (CG13463) can enhance acetyltransferase activity of Chameau, Flag-tagged EAChm (CG13463) gene product was bacterially expressed and purified using anti-Flag resin (Fig 3B). The enhancing activity of recombinant flag-tagged EAChm (rf-EAChm) was examined and compared with that of native EAChm. Acetyltransferase activity of native Chameau was enhanced by adding both rf-EAChm and native EAChm in a dose-dependent manner (Fig 3C). Thus, we concluded that *Drosophila* CG13463 gene product is EAChm and it stimulates acetyltransferase activity of native Chameau.

### Chameau and EAChm stimulates transcription *in vitro*

**Fig 4:**
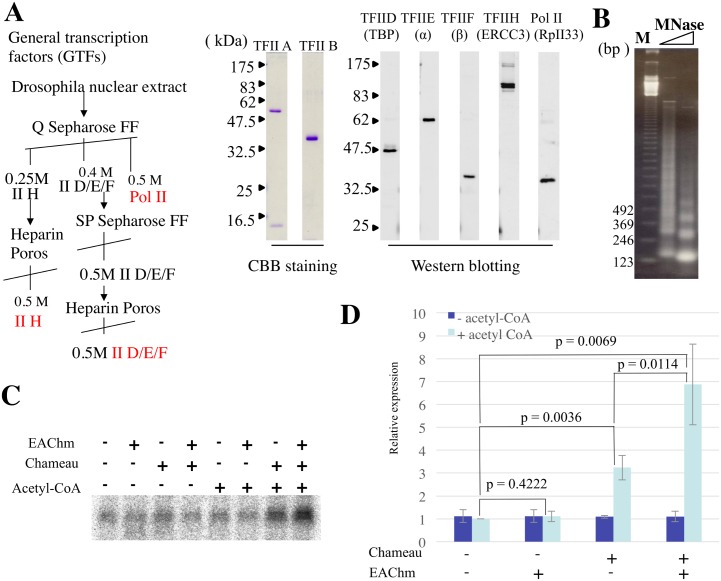
Chameau and EAChm stimulates transcription *in vitro*. A) Partial purification of GTFs. TFII D, E, F, H and RNA Polymerase II were evaluated by Western blotting. Bacterially expressed TFIIA and TFIIB were purified and evaluated by Coomassie brilliant blue R staining. B) Confirmation of salt-dialyzed chromatin assembly by Micrococcal nuclease (MNase) assay. Salt-dialyzed chromatin was partially digested by two steps diluted Micrococcal nuclease. Digested and extracted DNA was run on a 1.5% agarose gel and visualized by ethidium bromide staining. Lane M:123 bp DNA Ladder (Thermo Fischer scientific). This marker indicates multiples of 123bp (123-3075bp) C) Gel image and D) quantified graph of *in vitro* transcription assay. Salt-dialyzed chromatin was incubated with the combination of native Chameau (Glycerol gradient fraction 4) and recombinant EAChm in addition to the EcR/USP heterodimer, 0.7 mM acetyl-CoA, 3 mM ATP and the 20-hydroxy-ecdysone ligand. After incubation, the chromatin template was subjected to transcription by adding partially purified GTFs, recombinant TFIIB and TFIIA. Transcripts were detected by primer extension. Activation above basal transcription (+ actyl-CoA, - chameau, - EAChm) was estimated by a phosphorimaging analyzer. Error bars represent mean ±SD, n = 4. *p*-value of two-tailed Student’s t-test is indicated.

Acetyltransferase Chameau is known as the *Drosophila melanogaster* homologue of human Hbo1 and belongs to the Hbo1 subfamily of the MYST family of histone acetyltransferases [12]. Although most of the MYST family acetyltransferases are reported to be involved transcriptional activation, Chameau has been shown to function in transcriptional repression [14]. On the other hand, it has also been demonstrated that activating the JNK pathway stimulates the HAT function of Chameau, promoting histone H4 acetylation and targets gene transcription [15]. To clarify the function of Chameau and EAChm, we examined whether histone H3 acetyltransferase activity, composed of Chameau and EAChm, affects gene transcription from a chromatin template *in vitro*. For the transcription reaction, we used Drosophila enhancer with EcR/USP as an activator [16]. We partially purified general transcription factors (GTFs) and Pol II from the nuclear extract of *Drosophila* embryos (Fig 4A) and expressed TFIIA and TFIIB in E.coli and used them for *in vitro* transcription using salt-dialyzed chromatin as a template (Fig 4B). We used glycerol gradient fraction 4 as native Chameau and E.coli expressed recombinant EAChm. We confirmed that no other HAT was contained in this glycerol gradient fraction 4 without Chameau being present by using MS analysis. Recombinant EAChm had no effect on transcriptional activation by itself (Fig 4C and 4D). Chameau activated transcription 3.2 fold only in the presence of acetyl-CoA. Chameau and EAChm activated transcription 6.8 fold cooperatively and their activation is acetyl CoA dependent. Contamination of background HAT activity that affects transcription can be ignored (Fig 4C and 4D).

### Whole-transcriptome sequencing analysis shows overlap of Chameau and EAChm target genes *in vivo*

**Fig 5:**
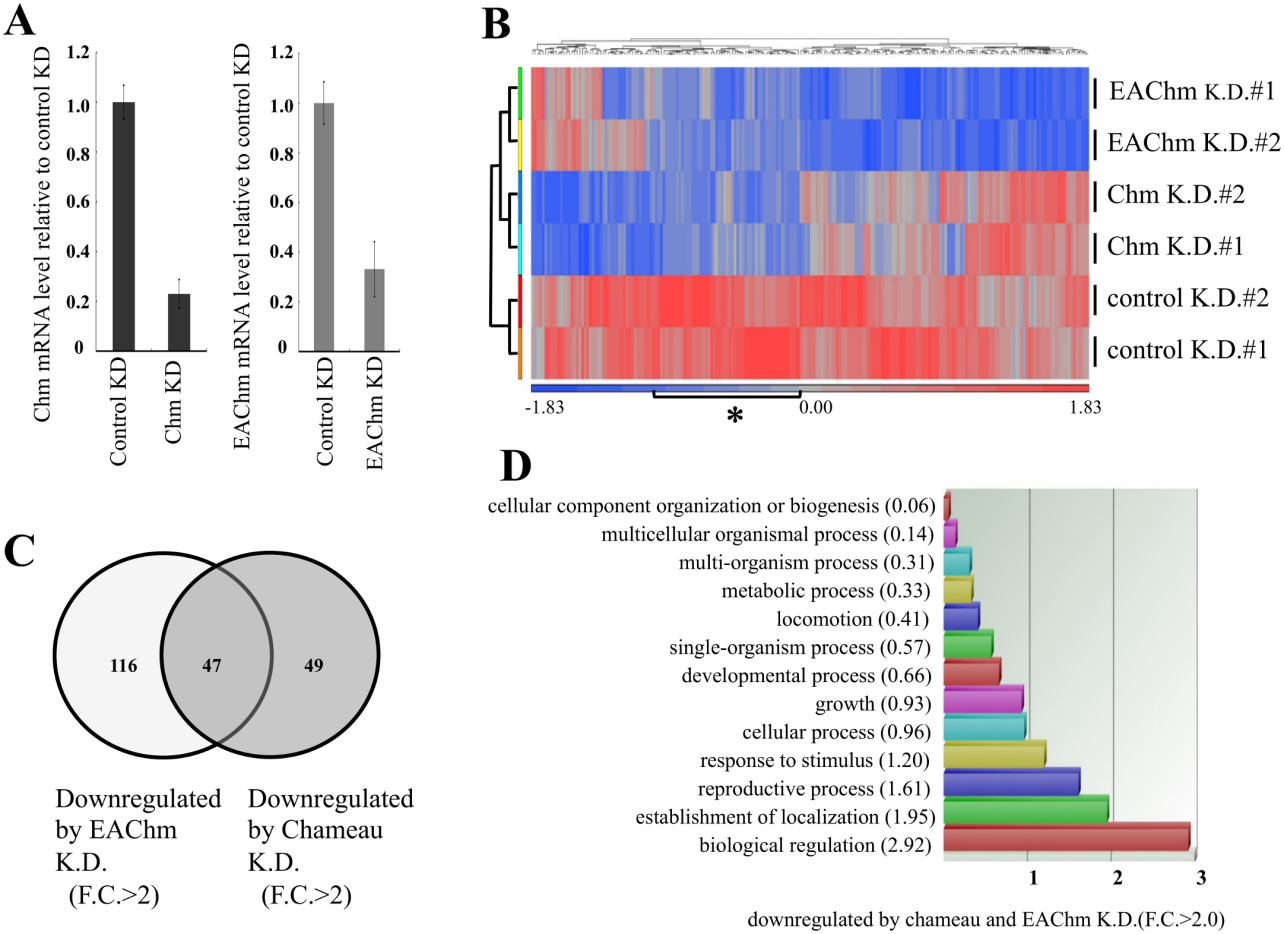
Chameau and EAChm target genes overlap *in vivo*. A) Evaluation of RNA interference by RT-qPCR. RT-qPCR was performed to document the efficiency of Chameau and EAChm KD. Expression level was normalized with reference to GAPDH. Error bars represent mean ±SD, n = 3. B) Heat map of downregulated genes in control knockdown (KD), Chameau KD and EAChm KD S2 cells. Blue and red indicate down and up regulated genes, respectively. The asterisk indicates the genes commonly downregulated by both Chameau and EAChm KD. C) Venn diagram of genes downregulated more than 2 fold (*p*-value < 0.05) by Chameau KD or EAChm KD compered to control KD. This diagram shows 47 genes that were overlapping as both Chameau and EAChm target genes. D) Gene ontology (GO) classification analysis of 47 genes common to both Chameau and EAChm KD at Fig 4C.

To find out the association between Chameau and EAChm in *vivo*, we performed RNA-seq analysis of drosophila S2 cells after Chameau or EAChm had been knocked down. Decreased Chameau and EAChm expression by RNA interference was confirmed by RT-PCR (Fig 5A). mRNA quantification was performed using transcript-level and a significant signal was filtered by including at least one of the signal having more than one reads per kilobase of exon per million mapped reads (RPKM). A scatter plot shows the correlation between duplicate samples (S1 Fig). Histone acetylation is generally considered to be associated with transcriptional activation. We focused on downregulated genes since we knocked down (KD) acetyltransferase activity. The Expression profile was analyzed by Clustering with 212 genes that decreased more than 2 fold (p-value < 0.05) by Chameau KD or EAChm KD compered to control KD (Fig 5B). There are some correlations between Chameau KD and EAChm KD as indicated by the asterisk in the heat map (Fig 5B) A Venn diagram was made composed of 163 genes, the expression of which were decreased by EAChm KD more than 2 fold (p-value < 0.05) and 96 genes which decreased by Chameau KD more than 2 fold (p-value < 0.05) compared to control KD. Forty seven genes among them are commonly down regulated by both Chameau KD and EAChm KD (Fig 5C, S1 File). Gene ontology (GO) classification of the 47 overlapping genes downregulated is shown in Fig 5D. To the contrary, directly or indirectly, some genes are upregulated by Chameau KD or EAChm KD. Clustering indicated that there was some overlapping of genes upregulated by Chameau KD and EAChm KD, however they were fewer than those overlapping genes downregulated by their knockout. Genes commonly up regulated by both Chameau KD and EAChm KD are indicated by the asterisk in the heat map (S2 Fig). A Venn diagram of upregulated genes also shows 29 genes that were overlapping as both Chameau and EAChm target genes, but fewer than that of downregulated genes (S3 Fig, S2 File). These results suggest that Chameau and EAChm regulate transcription of common genes *in vivo*.

## Discussion

In this study, we identified unique mechanisms showing that EAChm stimulates the MYST family histone Chameau acetyltransferase and activates transcription. Previously, the Hbo1/Chameau subfamily of the MYST family histone acetyltransferases has been mainly considered to function in an epigenetic mechanism of transcriptional repression since haploinsufficiency of Chameau leads to defects of position effect variegation [14]. Recently, it was also shown that Chameau cooperates with JNK signaling to promote transcriptional activation [15] and Hbo1, the human homologue of Chameau, is required for H3K14 acetylation and normal transcriptional activity during embryonic development [17]. These contradictory observations can be explained by either multiple targets or a co-regulator of Chameau. Our observation that EAChm stimulates Chameau acetyltransferases suggests the latter mechanism that a multiple co-regulator of Chameau plays a role in transcriptional regulation is correct.

As a co-activator that works together with HAT, chromatin remodeling factor is previously known. For example, it has been reported that NURF and histone acetylation by p300 synergistically facilitate transcription of chromatin [18]. However, it is not proven that NURF by itself facilitates histone acetylation. Furthermore, it has been reported that p300 acetylates nucleosomal core histones in the presence of ACF and an activator [19]. It was also not shown that ACF by itself could stimulate acetylation by p300. In this study, we have clearly shown that EAChm stimulates acetylation activity of Chameau by itself. Although, EAChm does not have a helicase domain, it is rich in acidic amino acids and may behave as a histone chaperon. Acidic regions have been identified in several histone chaperones and are thought to be the important for association with the positively charged residues of core histone. For example, nucleoplasmin, NAP-1, Asf1 and FACT are rich in acidic residues and can associate with positively charged core histones [20–23]. Therefore, EAChm which is rich in acidic residues may act like a histone chaperone. On the basis of these facts, we speculate that EAChm is a histone chaperone and stimulates acetylation activity of Chameau by histone transfer or eviction.

To find out if an association between Chameau and EAChm in *vivo* exists, we performed RNA-seq analysis of S2 cells after knocking down of Chameau and EAChm. Hierarchical clustering and Venn diagram analysis show some overlapping target genes. However, it might be an indirect effect. Therefore, further research is needed to complement *in vitro* results by *in vivo* studies.

A previous study reported that the knockdown of CG13463 showed lethality at the early pupa stage [24], and the life-cycle time-course expression profiling of *D*. *melanogaster* shows that the expression of CG13463 is high and specific for testis and salivary glands in adults, imaginal disc in wandering L3 larvae and in the 16–24 hr embryo stage (see FlyBase report; http://flybase.org). These observations suggest that EAChm plays an essential role, possibly by enhancing histone acetylation to advance the development at a specific stage in the development of *Drosophila*.

## Materials and Methods

### Purification of histone H3 acetylation activity

Histone H3 acetylation activity was purified from *Drosophila* embryos that were collected from 0 to 12 h after egg deposition, as shown in Fig 1A. Drosophila nuclear extract from 2 kg of embryos were prepared as described by Kamakaka and Kadonaga (1994)[25]. In the final step of this method, nuclei were separated into soluble and insoluble pellet fractions by ultracentrifugation. A pellet fraction was extracted by adding KCl to a final concentration of 0.52 M. Then, the supernatant extracted with 0.52 M KCl was subjected to ammonium sulfate precipitation. Protein precipitated at 10% (NH_4_)_2_SO_4_ was removed and the pellet precipitated at 60% (NH_4_)_2_SO_4_ was dissolved and dialyzed against 0.1 M KCl HEG buffer [25 mM HEPES (pH7.6), 0.1 mM EDTA, 10% glycerol, 0.01% NP-40, 0.2 mM PMSF and 1 mM benzamidine]. The dialyzed sample was applied to a column of 120 ml of SP sepharose F.F. (Pharmacia) and eluted stepwise by 0.25 M KCl, 0.4 M KCl and 1.0 M KCl HEG buffer. H3 acetylation activity was confirmed by HAT assay (described below). H3 acetylation activity was mainly eluted in the 0.4 M KCl fraction. Then, the 0.4 M KCl fraction was diluted to 0.1 M KCl HEG. The Diluted sample was subsequently subjected to 120 ml Q sepharose F.F(Pharmacia) column chromatography and eluted by a 10 column volume linear gradient from 0.1 M to 1.0 M KCl HEG buffer. H3 acetylation activity was mainly eluted in the 0.25 M KCl fraction. The 0.25 M KCl fraction was then diluted to 0.1 M KCl HEG. The diluted sample was subjected to 8 ml POROS Heparin (Roche) column chromatography and eluted by a 20 column volume linear gradient from 0.1 M to 2.0 M KCl HEG buffer. H3 acetylation activity was mainly eluted in the 0.8 M KCl fraction. The 0.8 M KCl fraction was then diluted to 0.1 M KCl HEG. Whereupon the diluted sample was subjected to 2 ml Blue sepharose F.F. (Pharmacia) column chromatography and eluted using a 20 column volume linear gradient from 0.1 M to 2.0 M KCl HEG buffer. We repeated this Blue sepharose chromatography step three times. During the first Blue sepharose chromatography step, H3 acetylation activity was mainly eluted in the 0.9 M KCl fraction. After which the 0.9 M KCl fraction was diluted to 0.1 M KCl HEG. Whereupon the diluted sample was subjected to a second Blue sepharose chromatography step and eluted in 0.8 M KCl HEG. At the third Blue sepharose chromatography step, H3 acetylation activity was mainly eluted in the 1.0 M KCl fraction and dialyzed against 50 mM KCl HEG. The dialyzed fraction was then subjected to 20–50% glycerol gradient sedimentation (BECKMAN SW60 rotor, 60,000 rpm for 20 hours at 4°C). Active fractions from the glycerol gradient sedimentation were analyzed using a Thermo LCQ ion trap mass spectrometer.

### Mass-spectrometry

The silver staining gel of the glycerol gradient sedimentation was excised and destained in 15 mM Potassium ferricyanide/ 50 mM Sodium thiosulfate for 10 min with shaking. The gel was washed three times with distilled water and once with 200 mM NH_4_HCO_3_. The gel was dried and reduced in 10 mM DTT/ 25 mM NH_4_HCO_3_ at 50°C for 1 hr. Subsequently, the gel was alkylated in 27.5 mM iodacetoamido/ 25 mM NH_4_HCO_3_ at room temperature for 20 min. The alkylated gel was washed with acetonitrile, 25 mM NH_4_HCO_3_ and with acetonitrile again. Protein in the gel was digested with 10 μg/μl Modified Trypsin (Promega) / 50 mM NH_4_HCO_3_ overnight at 37°C. Digested peptides were eluted by adding 0.05% formic acid/distilled water. Trypsin digested peptides were analyzed by reversed-phase liquid chromatography on an EASY-nLC 1000 system coupled with a Thermo LCQ ion trap mass spectrometer (Thermo Fischer Scientific). Peptides were loaded on to a NANO HPLC CAPILLARY COLUMN (20cm-75micron ID, Nikkyou Technos, Japan) and eluted directly into a mass spectrometer (spray voltage: 1.5 kV, capillary temperature: 200°C) using a linear gradient (0–35% buffer B 60 min, 35–100% buffer B 10 min) of acetonitrile at a flow rate of 300 nl/min (Buffer A: 0.05% Formic acid, 99.95% H_2_O, Buffer B: 0.05% Formic acid, 99.95% acetonitrile). MS/MS data were acquired in a data-dependent mode. Collected data were analyzed using the SEQUEST program for peptide identification.

### Expression and purification of recombinant EAChm

Flag-tagged EAChm was subcloned into a pET vector generating pET- EAChm. Flag-tagged EAChm was produced in E. coli strain Rosseta (DE3) pLysS (Invitrogen). Lysate of E. coli in which flag-tagged EAChm was expressed was immunopurified with flag M2 agarose (Sigma). After extensive washing of the agarose, flag-tagged EAChm was eluted with flag peptides (Sigma) and dialysed against 50 mM KCl. The purified recombinant EAChm was subjected to 10% sodium dodecyl sulfate-polyacrylamide gel electrophoresis (SDS-PAGE) and then visualized using Coomassie brilliant blue R staining.

### Chromatin assembly and Histone acetylation assay

Chromatin was made by mixing the pEcE4 DNA template containing five ecdysone response elements (EcRE) upstream of the adenovirus E4 promoter and core histone octamer in the presence of high salt concentration and subsequent salt dialysis. The resulting chromatin was centrifuged on a linear 15 to 40% glycerol gradient for further purification. For the acetylation assay, the incubation temperature of the whole interaction was performed at 27°C. At first, a 1 μl aliquot of chromatin containing 62.5 ng of plasmid DNA and 62.5 ng of histones was mixed with 3 mM ATP and 2 mM MgCl_2_, and then various active fractions from column purification were added as indicated. After 20 min, 6.67 mM Na(C3H7COO), 7.4 kBq [3H]acetyl-CoA, and 50 mM KCl HEG were added in a final volume of 7.5 μl. The reaction was incubated at 27°C for 2.5 h and analyzed on 13.5% SDS—PAGE with subsequent fluorography basically as described previously [22, 26].

### Purification of general transcription factors and recombinant proteins for *in vitro* transcription

Drosophila nuclear extract from 0- to 12-h-old embryos (200 g) was applied to a 20 ml Q Sepharose FF column and the bound protein was eluted with a linear KCl gradient (10 column volumes) from 0.1 to 1 M KCl in HEG. General transcription factors were confirmed by Western blotting. TFIIH and TFIIA were eluted in the 0.25 M KCl fraction. TFFIID, TFIIE and TFIIF were eluted in the 0.4 M KCl fraction. RNA polymerase II was eluted in the 0.5 M KCl fraction. TFIIH was subjected to 2 ml POROS heparin column chromatography and eluted with a linear KCl gradient (10 column volumes) from 0.1 to 1 M KCl in HEG. TFIIH was eluted in the 0.5 M KCl fraction. TFFIID, TFIIE and TFIIF were subjected to 2 ml SP sepharose FF column chromatography and eluted with a linear KCl gradient (10 column volumes) from 0.1 to 1 M KCl in HEG. TFFIID, TFIIE and TFIIF were eluted in the 0.5 M KCl fraction. TFFIID, TFIIE and TFIIF were further purified with 1ml POROS heparin column chromatography. TFFIID, TFIIE and TFIIF were eluted in the 0.5 M KCl fraction. Bacterially expressed His-tagged TFIIB was purified by Ni-NTA affinity chromatography and Flag-tagged TFIIA was purified by flag M2 agarose. Purification of general transcription factor and recombinant proteins were basically performed as described previously [16].

### *In vitro* transcription assay

Chromatin template assembled by salt dialysis, containing five ecdysone response elements (EcRE) upstream of the adenovirus E4 promoter, was employed for the *in vitro* transcription assay in which partially purified Drosophila general transcription factors and recombinant TFIIB were used basically as described previously [16]. The chromatin template was transcribed by adding GTFs and factors as indicated and the RNA products were evaluated by primer extension analysis. The experiment was performed four separate times to ensure reproducibility.

### Cell culture and RNA interference

S2 cells were grown in Schneider’s Drosophila media (Gibco) with 10% FBS. Double-strand RNAs (dsRNA) corresponding to portions of the Chm, EAChm, and EGFP (enhanced green fluorescent protein, as a control) were synthesized using the MEGAscript RNAi kit (Thermo Fischer Scientific). 25 μg of dsRNA was added to 8x 10^6^ cells/ 3.5 cm dish in serum free Schneider’s Drosophila media for 6 h. The cells were subsequently had their medium changed to Schneider’s Drosophila media with 10% FBS and cultured at 26°C for 72 h.

The primer sequences for generation of dsRNA were as follows;

Chm Forward,

5’- TAATACGACTCACTATAGGGTACGCCTTGGAGTCCGGCAGC -3’,

Chm Reverse,

5’- TAATACGACTCACTATAGGGTACAGGGGAACCTTCCGCCCG -3’,

EAChm Forward,

5’- TAATACGACTCACTATAGGGTACGCAATCCATAGCATTTGCTC -3’,

EAchm Reverse,

5’- TAATACGACTCACTATAGGGTACCGATGTCATCTAGAACCTGG -3’,

EGFP Forward,

5’- TAATACGACTCACTATAGGGTACATGGTGAGCAAGGGCGAGGAG -3’,

EGFP Reverse,

5’- TAATACGACTCACTATAGGGTACTTACTTGTACAGCTCGTCCAT -3’

For knockdown, S2 cells were treated with dsRNA for 3 days.

### RNA extraction and reverse transcription (RT)

Total RNA was extracted from S2 cells treated with dsRNA by ISOGEN II (NIPPON GENE). The amount of 0.5 μg of total RNA was used for RT reaction using random hexamers (TAKARA), oligo(dT) primer (Life Technologies), and M-MuLV reverse transcriptase (NEB) for 1 hour at 42°C. RNA quantification by real-time PCR was performed using the KAPA SYBR FAST qPCR Kit (Kapa Biosystems), and normalized with reference to GAPDH. The primer sequences for RT-qPCR were as follows;

GAPDH Forward, 5’- ACCGACTTCTTCAGCGACACC -3’

GAPDH Reverse, 5’- GCTCTGCATATACTTGATCAG -3’

Chm Forward, 5’- AGCACCCTTCAAGCTCTCGG -3’

Chm Reverse, 5’- CTTAAGCAGGAGTCGTCGATC -3’

EAChm Forward, 5’- CATAGTCTCAGTTGCACTCAG -3’

EAChm Reverse, 5’- CAAATTCATCTGGGGAGCTGC-3’.

### RNA-seq

Quality of total RNA was checked using the Agilent RNA 6000 Nano kit with an Agilent 2100 Bioanalyzer instrument (Agilent Technologies). A library was prepared using the TruSeq Stranded mRNA LT Sample Prep kit (Illumina) and sequenced with the MiSeq system (Illumina). Samples were sequenced to a depth of approximately 4 million uniquely mapped reads per sample. Sequences were aligned to the Drosophila *melanogaster* dm3 reference genome with the program of Miseq, allowing one mismatch. Reads that could be uniquely mapped to a gene were used to calculate the expression level. The level of gene expression was quantified by the number of uniquely mapped reads per kilobase of exon per million mapped reads (RPKM). Scatter plots were created using the Partek Genomics Suite to evaluate the correlation between the duplicate sample data sets. Gene ontology (GO) analysis was performed using a Partek Genomics Suite. Significantly enriched GO functional groups were defined as having an enrichment score equal to or greater than 3 (P value < 0.05), and each functional group was assigned with a GO enrichment score calculated using Fisher`s exact test. All RNA-seq data can be found online in the NCBI GEO SuperSeries GSE73098.

### Heat map construction and hierarchical clustering

Heat map and hierarchical clustering of the gene expression profiles were created using the Partek Genomics Suite. Heat maps of control KD, Chameau KD and EAChm KD were normalized by calculating the RPKM based on the sum of all reads found in the exon regions of that gene. To identify differentially expressed genes, one-way analysis of variance (ANOVA) was used. By setting P < 0.05 and fold-change (FC) settings FC > 2, we obtained lists of differentially expressed genes between control KD and Chameau KD or control KD and EAChm KD cells. Hierarchical clustering of control KD, Chameau KD, and EAChm KD cells was performed using 212 genes that varied significantly among each of the groups with a statistical P value < 0.05. We subtracted the mean of the gene expression levels in the six paired samples, normalized each row in the data table, calculated the distance using Pearson correlation, and then used a “pairwise average-linkage” hierarchical clustering method for clustering. The scale was the standardized RPKM value.

### Statistical analysis

The statistical analysis was performed using Excel 2013 software (Microsoft). Comparison were performed with two-tailed Student’s *t*-test. The error bars indicate the standard deviation of four independent experiments.

## Supporting Information

S1 Fig Scatterplot of gene expression levels between RNA-seq duplicate samples. (TIFF)

S2 Fig Heat map of upregulated genes in control, Chameau and EAChm KD S2 cells. Asterisk indicates the upregulated genes common to Chameau and EAChm KD.
(TIFF)

S3 Fig Venn diagram of genes more than 2 fold upregulated (*p*-value < 0.05) by Chameau or EAChm KD compared to control KD. (TIFF)

S1 File List of commonly downregulated 47 genes by both Chmeau KD and EAChm KD. (XLS)

S2 File List of commonly upregulated 29 genes by both Chmeau KD and EAChm KD. (XLS)
