# Supplementary figures and images for: Enhancer of Acetyltransferase Chameau (EAChm) Is a Novel Transcriptional Co-Activator
Source: PLoS One. 2015 Nov 10;10(11):e0142305. doi: 10.1371/journal.pone.0142305 (PMC4640846; doi:10.1371/journal.pone.0142305)

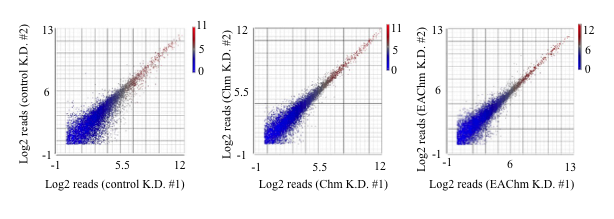

Supplement: S1 Fig — (TIFF) [file pone.0142305.s001.tiff]

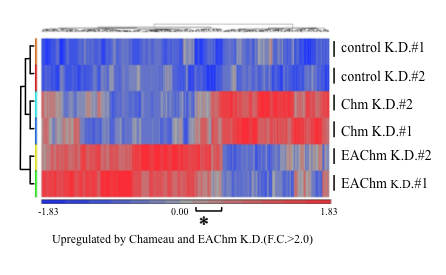

Supplement: S2 Fig — Asterisk indicates the upregulated genes common to Chameau and EAChm KD. (TIFF) [file pone.0142305.s002.tiff]

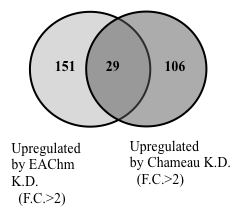

Supplement: S3 Fig — (TIFF) [file pone.0142305.s003.tiff]
